# Behavioural and Neurophysiologic Features of State Dissociation: A Brief Review of the Literature and Three Descriptive Case Studies

**DOI:** 10.3233/ben-2009-0248

**Published:** 2010-06-29

**Authors:** Alberto Raggi, Filomena I.I. Cosentino, Bartolo Lanuzza, Raffaele Ferri

**Affiliations:** Department of Neurology I.C.Oasi Institute for Research on Mental Retardation and Brain Aging (IRCCS)TroinaItaly

**Keywords:** Agrypnia excitata, dissociated states of wakefulness and sleep, fatal familial insomnia, Mulvihill-Smith syndrome, narcolepsy, REM sleep behaviour disorder

## Abstract

Wakefulness, rapid eye movement (REM) and non-REM sleep are not always mutually exclusive conditions, as commonly assumed. In some cases, the declaration of any state may be incomplete and states can fluctuate rapidly, resulting in peculiar behavioural syndromes such as narcolepsy, REM sleep behaviour disorder and status dissociatus. We briefly introduce this topic and discuss three suggestive clinical cases.

## 1. Introduction

Three states of being can be recognised: wakefulness, rapid eye movements (REM) sleep, and non-REM (NREM) sleep; each of them has its own neuroanatomic, neurophysiologic and neurochemical substrates [25].

In the same sleep cycle, subsequent stages of NREM sleep are characterized by progressively slower frequencies and higher voltage activities and correspond to progressively deeper states of sleep. Stage 1 is light sleep where people drift in and out of sleep and can be awakened easily; in this stage, the eyes move slowly and muscle activity slows down. During this stage, many people experience sudden muscle contractions preceded by a sensation of falling (hypnagogic phenomena). In stage 2, eye movements stop and brain waves become slower with only occasional bursts of rapid potentials. In this stage it is possible to observe spindles (12–16 Hz waves that usually last for 0.5 to 1.5 seconds) and K-complexes (brief high-voltage peaks, usually greater than 100 $\mu$V, that last for more than 0.5 seconds). During the last stage of NREM sleep, also called slow-wave sleep (SWS) because of the high-voltage and slow-frequency electroencephalographic (EEG) signal, the muscles are relaxed, heart rate and blood pressure decline and gastrointestinal motility increases. Ideally, approximately 90 minutes after sleep onset, several physiological changes occur. The EEG becomes desynchronized, showing a low-voltage, fast-activity pattern similar, even if not identical, to that of the waking state. This brain pattern is coupled with almost complete loss of muscle tone. Body temperature suddenly begins to change in the direction of the environmental temperature and pupils become myotic because of a broad suppression of sympathetic activity. Reduced homeostasis is also a fundamental property of this sleep stage. There is an association between the desynchronized EEG and the presence of rapid eye movements monitored by electrooculogram (EOG) [8]. This active sleep stage has consequently been called *REM sleep*. Finally, most subjects awakened from REM sleep readily recall dreaming [25].

ISSN 0953-4180/09,10/$27.50 © 2009/2010 – IOS Press and the authors. All rights reserved

During a typical night sleep, the normal adult individual alternates between periods of NREM sleep and REM sleep, with REM stages recurring at somewhat regular intervals four to six times each night. In total, REM sleep occupies approximately 20–25% of the sleep time of young adults. NREM sleep stage 2 occupies about one-half of total sleep time, while SWS about 15% [18].

When behavioural, polygraphic and cellular variables occur in substantial synchronization, a state, as described above, becomes fully declared. Nevertheless, these conditions are not always mutually exclusive [30,31,33,35].

Evidence for only partly defined or mixed states of being is available both from animal experimentation [7, 9,11,19,20,24,38,40,60,61] and from human clinical studies [30].

Furthermore, numerous studies have replicated the finding of mentation not only in REM, but also in NREM sleep. About 50% of subjects appear to have noticeable degraded recall of mentation from NREM sleep [14,49,53]. Two different theoretical models have been proposed to account for this finding: a one-generator model, in which mentation is generated by a single set of processes regardless of physiological differences between REM and NREM sleep [1,10] and a two-generator model, in which qualitatively different generators produce cognitive activity in two states [6, 21,22,55]. One possible reconciliation theory taking into account both the one- and the two-generator models, is that sleep mentation is tightly coupled to REM sleep processes, but that some of these processes under certain circumstances may dissociate from REM sleep and stimulate mentation in NREM sleep in a covert fashion [46]. "Covert" REM sleep is thus defined to be "any episode of NREM sleep for which some REM sleep processes are present, but for which REM sleep cannot be scored with standard criteria" [46]. Covert REM sleep is suggested by the concept of "intermediate sleep" introduced by Lairy et al. [26] who were among the first to identify atypical mixtures of REM/NREM in human subjects. Their notion of "intermediate sleep" was of a sleep that typically arises between REM and NREM sleep episodes but that consists of elements of both.

According to Mahowald and Schenck [30,31] areas of overlap among states can be classified in:

1. Wake/NREM combination
   - A. Disorders of arousal such as sleepwalking, sleep terror, confusional arousals,
   - B. Psychogenic dissociation;
2. Wake/REM combinations
   - A. Cataplexy, hypnagogic hallucinations, sleep paralysis,
   - B. REM sleep behaviour disorder (RBD),
   - C. Lucid dreaming,
   - D. Delirium;
3. Wake/NREM/REM combinations
   - A. Status dissociatus,
   - B. "Parasomnia overlap" syndromes;
4. NREM/REM combinations that is theoretically possible, but not accompanied by conscious awareness.

We describe the behavioural and neurophysiologic aspects of three patients with state dissociation.

## 2. Case reports

### *2.1. Case 1: Two state boundary dyscontrol conditions may overlap*

A 24-year-old man was referred to the sleep clinic for evaluation of a 7-year history of progressively severe, inappropriate excessive daytime sleepiness. His past clinical history was uneventful. The physical examination was normal. The patient was drug-free at the time of our first evaluation and had never been previously treated with antidepressant. Genotyping was positive for HLA-DQB1*0602 allele.

*Behavioural and neurophysiologic features*

He stated he could fall asleep any time, also in inappropriate circumstances and places. His attacks of irresistible desire to sleep usually occurred suddenly and were of brief duration. A sudden loss of postural tone with the risk to crumple to the ground was also reported. Emotion, laughter or crying, could precipitate an attack. Vivid dreams or hallucinations occurred as the patient fell asleep or, occasionally, when apparently awake. On awakening, he was unable to move for 2–3 minutes. In association to this clinical context, clearly resembling that firstly described by Gélineau [15], there was the presenting complaint of some vigorous sleep behaviours such as throwing punches and displaying nonsense vocalizations.

The cognitive status was normal; his Epworth sleepiness scale score [45] was 17/24. A multiple sleep laten-

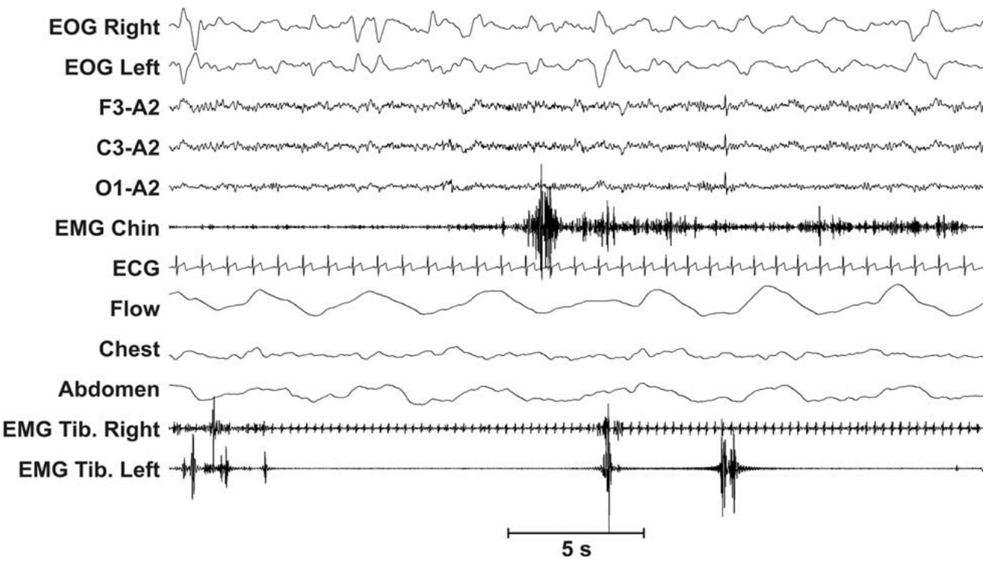

Fig. 1. Polysomnogram of REM sleep without atonia in our narcoleptic patient with REM sleep behaviour disorder. Normally during REM sleep there is atonia of all somatic muscles except the diaphragm. This recording shows prominent tonic and phasic EMG activity of the chin and excessive twitching of the legs, during an epoch of REM sleep.

Table 1
Sleep scoring parameters of patient 1

| | |
|---|---|
| TIB, min | 545.0 |
| SPT, min | 541.5 |
| TST, min | 433.0 |
| SOL, min | 1.5 |
| REM latency, min | 6.5 |
| SS/hour | 12.2 |
| AWN/hour | 7.5 |
| SE, % | 79.4 |
| WASO, % | 20.0 |
| S1, % | 5.3 |
| S2, % | 39.2 |
| SWS, % | 15.4 |
| REM, % | 19.1 |

cy test [52] was consistent with narcolepsy (mean sleep latency = 4.8 min and 3 out of 4 sessions characterized by sleep-onset REM periods).

The patient underwent polysomnography (Fig. 1) that revealed intense phasic and tonic muscle activity (chin and posterior tibialis) during REM periods accompanied by limb movements resembling REM sleep without atonia as usually seen in REM sleep behaviour disorder (RBD), as described by Schenck et al. [54]. Table 1 reports the sleep scoring parameters obtained in this patient which show the typical features of nocturnal sleep of narcolepsy (i.e. short sleep latency, short REM sleep latency, and increased sleep fragmentation, as indicated by the high number of stage shifts and wakenings).

### 2.2. Case 2: Fatal Familial Insomnia . . . "to chase sleep away"

The patient was a 63-year-old woman with sleep and behavioural features, family history, genetic pattern of Fatal Familial Insomnia (FFI) as reported previously [28,42]. A pathological verification was also performed after 14 months of clinical course that led her to death in a state of akinetic mutism and emaciation. Methods and clinical history are described in details in Raggi et al. [51].

*Behavioural and neurophysiologic features*

She started her clinical course with developing visual fatigue and after a few weeks she began presenting personality changes such as apathy, disinterest and depression. She was no longer able to nap or to fall asleep at night. She looked somnolent and perplexed during the daytime. She showed defective language comprehension and was constantly inattentive, slovenly, shy, fearful and perplexed. She was in a clouded state, apathetic with delayed responses. She followed the movements with her gaze or looked around as if she was searching for something that, very often, she tried to reach or touch with her hands.

During the months of the disease course, the patient developed a progressive motor impairment, consisting of spontaneous myoclonic bursts, dysmetria and disequilibrium. She had frequent episodes of dream enact-

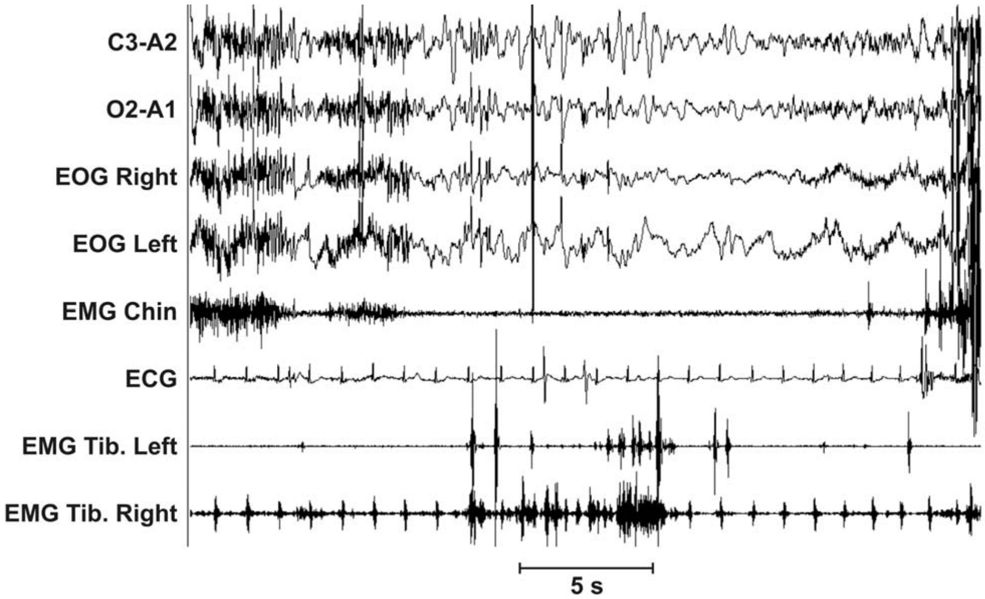

Fig. 2. Polysomnographic example of a sudden undifferentiated sleep attack. The presence of saw-tooth waves on central EEG, of tibialis anterior muscle twitches, together with the EMG flattening of the chin muscle tone are suggestive of REM sleep.

ment, whereby she displayed complex jerk-like movements, presumably mimicking the content of dreams. These oneiric stupor episodes occurred more often with open eyes. With progression of the disease, the patient became more and more confused, alternating between wakefulness and oneiric confusional states.

The electroencephalogram (EEG) showed global slowing of the cerebral electrical activity without lateralisation signs.

A video-polysomnographic study did not show any stage of stable sleep, but only short episodes (10–30 seconds) of EEG slowing associated, in some instances, with chin EMG atonia and phasic EMG activity recorded at the level of the tibialis anterior muscles (Fig. 2).

Total sleep time was 44 minutes, with 35 minutes in stage 1 and 6 minutes in slower NREM sleep. Episodes of possible REM sleep amounted to 3 minutes. The video showed also phases of gestural activity of the upper limbs and phasic activity of the lower limbs.

The wakefulness and sleep cycle were assessed by actigraphy for 5 nights. A virtual absence of sleep during the second night, and reduced duration (with relevant motor activity) in the other 4 nights were observed.

### 2.3. *Case 3: Mulvihill-Smith syndrome, a clinical condition where agrypnia excitata occurs*

The patient was a 25-year-old woman when evaluated by our team for the first time. She was without a family history for any behavioural neither sleep disturbances. She had a history of growth delay, relapsing respiratory system infections, pigmented lesions affecting trunks and limbs, bilateral sensory neural hearing impairment, squamous cell carcinoma of the tongue, premature menopause in accordance with a clinical phenotype of Mulvihill-Smith syndrome [2, 44,63]. Further details of this case and of instrumental procedures have been described previously [12].

*Behavioural and neurophysiologic features*

The patient was not well oriented in time, her gait was slightly ataxic and a fine postural tremor of both hands was also observed.

Neuropsychological testing disclosed the presence of mild cognitive impairment: a score of 20 (normal values $\geqslant 24$) was obtained at the Mini Mental State Evaluation [13] and of 56 at the Wechsler Adult Intelligence Scale-Revised Performance [62]. Mood was constantly depressed.

The clinical picture continued to worsen progressively and the patient used to show daytime fluctuations of her level of confusion and alertness with visual hallucinations. She died at the age of 26 years in a state of emaciation.

Routine EEG was uninformative because of the poor collaboration of the patient.

Two overnight video-polysomnographic recordings showed the absence of clear sleep episodes. The patient used to spend most of the time with closed eyes, desyncronized EEG activity, presence of eye movement similar to those recorded during wakefulness, irregular breathing, heart arrhythmia (Fig. 3A) and afinalistic movements of the upper limbs and hands which often simulated the movements needed to button up a shirt. More rarely, some short episodes were noticed, lasting for approximately 1 minute, which were characterized by some slow-wave mixed with desynchronised EEG activity, low chin muscle tone, presence of slow eye movements, heart arrhythmia, regular breathing (Fig. 3B) and absence of motor activity at the four limbs, during which the patient seemed to have poor contact with the environment. Finally, other fragments were characterised by the presence of a periodic respiratory pattern consistent with the regular occurrence of central apnea episodes, heart rate arrhythmia, desynchronised EEG activity, high chin muscle tone and presence of rapid eye movements (Fig. 3C). During these episodes the patient showed stereotyped afinalistic movements of the upper limbs and hands.

The patient seemed to have mentation associated with the motor activity during sleep; however she was not able to describe it accurately because of her compromised cognition and her speech deficit due to the tongue postsurgical state. Also the words pronounced during sleep were not completely intelligible.

## 3. Discussion

The first case had a clinical diagnosis of both narcolepsy with the characteristic tetrad (sleep attacks, cataplexy, sleep paralysis, hypnagogic hallucinations) and RBD.

The study of narcolepsy has been instrumental along the years in promoting the concept of state dissociation: the attacks represent a clear intrusion of REM sleep into wakefulness; the paralysis in morning is when the REM atonia persists after awakening (many children showing this phenomenon in normal/non pathological conditions are “blamed” by their parents that they do not want get up to go to school).

Narcolepsy has a clear genetic component: in fact over 90% of patients carry the HLA- DQB1*0602 allele. Despite a genetic component, the risk of a first-degree relative developing narcolepsy is only 1–2%, therefore the genetic component is neither necessary nor sufficient to cause the disease [3,39]. From the animal experiments it was possible to identify the relationship between hypocretin-1 or orexin (a neuropeptide confined to a small number of cells in the hypothalamus) and narcolepsy. It seems that patients with narcolepsy have lost the hypocretin-producing cells, possibly trough an immune-mediate mechanism [57]. This peptide has an excitatory influence on the histaminenergic, monoaminenergic, cholinenergic systems in the brainstem and diencephalon. Consequently, the lack of the excitatory influence may cause a reduction of the thalamo-cortical arousal [48] and therefore an irresistible need to fall asleep.

RBD is a parasomnia predicted by animal experiments long time ago [24]. The typical complaint of a patient with RBD is violent dream-enacting behaviour that is potentially injurious to the individual or bed partner. These behaviors include talking, yelling, swearing, punching, kicking, jumping, or running out of the bed. The violence of the sleep behaviors is often discordant with waking personality. Some patients adopt extraordinary measures to avoid injury, such us putting mattresses on the floor and removing any furniture from the bedroom.

There are acute and chronic forms of RBD. The acute form is almost always induced by medications (monoamine oxidase inhibitors, tryciclic antidepressants, SSRIs, venlafaxine) or associated with alcohol, barbiturate, meprobamate withdrawal [35]. The chronic form of RBD is usually either idiopathic or associated with neurological disorders. There is growing evidence of its association with neurodegenerative disorders, particularly the synucleinopathies. RBD represents, in many cases, the first manifestation of these conditions. RBD, in fact, may precede any other clinical manifestation of the synucleinopathies by more than ten years [4].

In man the structure analogous to the subcoeruleus region of cat [24] and sublaterodorsal nucleus of rat [27] is proposed to be the nucleus (and its associated efferent and afferent pathways) crucial to RBD pathophysiology [5]. When its inhibitory function on the magnocellular reticular formation, which is responsible for normal skeletal muscle atonia during REM sleep, is compromised, prominent motor activity accompanies dreaming permitting the acting-out of dream mentation [35].

Both narcolepsy and RBD represent state boundary dyscontrol conditions [35], therefore it should be no surprise that there is a high incidence of RBD in patients with narcolepsy [47]. So it was in the case described here.

The second patient described had a clinical diagnosis of FFI. Imaging and pathological studies showed

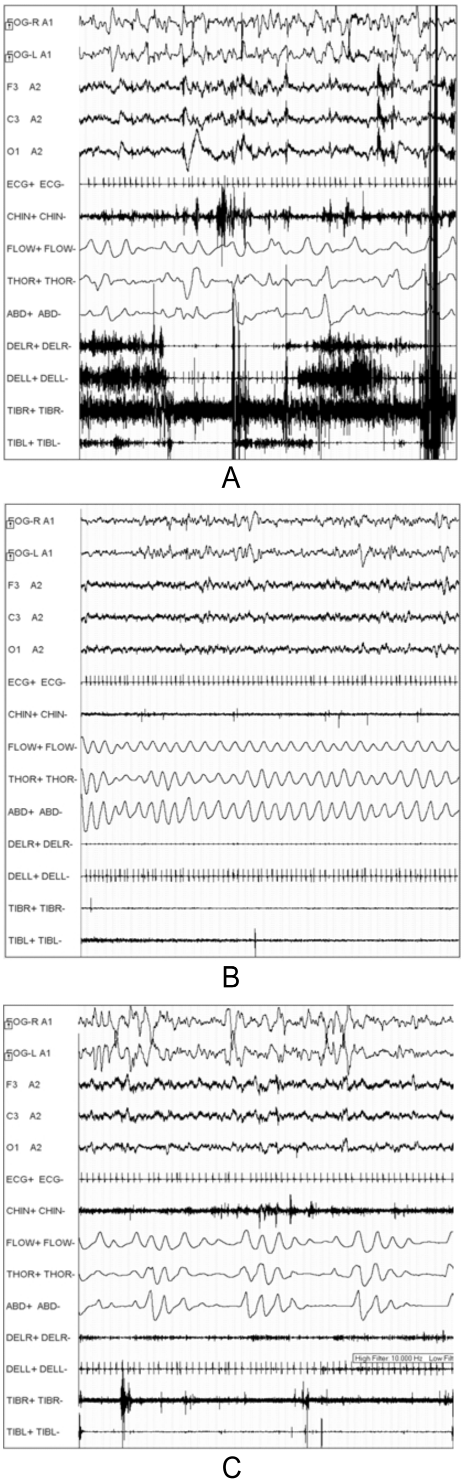

Fig. 3. Patient 3. **A**. Polysomnographic recording showing desynchronized EEG activity, presence of eye movements similar to wakefulness, irregular breathing, heart arrhythmia. **B.** Desynchronized EEG activity with mixed slow waves, low chin muscle tone, slow eye movements, heart arrhythmia and regular breathing. **C.** Periodic respiratory pattern characterized by recurrent central apnoea episodes, heart arrhythmia, desynchronized EEG activity, high chin muscle tone and presence of rapid eye movements.

prominent thalamus pathology. The patient appeared to be either awake or in NREM sleep. She used to experience vivid dreaming (REM sleep phenomenon) and to display frequent muscular twitching (REM sleep phenomenon), and yet she had no polygraphic features of either REM or NREM sleep.

The thalamus plays a fundamental role in SWS generation [23]. The dorsomedial (DM) and anterior (A) thalamic nuclei are interposed in the circuitry connecting the limbic areas to the hypothalamus and the basal forebrain. The DM nucleus represents an intermediate station in the trans-basal ganglionic circuit of the limbic system linking the ventral striatum and pallidum with the prefrontal cortex. Studies in the cat demonstrate that the DM nucleus receives inputs, with direct GABAergic projections, from the hypothalamus [17] and the basal forebrain [59]. Moreover, the medial thalamus contributes in setting the sympathetic tone via hypothalamic neurons [42]. In fact, bicuculline injections in the DM cause hypertension and tachycardia through removal of GABAergic inhibition, reproducing some autonomic features of FFI [56].

Clinical and neurophysiologic patterns similar to those described in this case can be seen in Morvan's disease and in delirium tremens, which combine lack of NREM, especially SWS, with oneirism, prominent autonomic activation and severe motor agitation [50]. Both conditions are characterised, as FFI, by thalamic involvement. The term *Agrypnia Excitata* has been suggested [29,43] to account for the association of SWS loss (*agrypnia* that means *to chase sleep away*) and abnormal REM sleep (*excitata*). The abnormalities of this condition seem to be underscored by dysfunctions within the thalamo-limbic circuits [58]. Moreover, *Agrypnia Excitata* may also be categorized as a particular instance within the broader concept of *Status Dissociatus*, as primarily defined by the presence of ambiguous, multiple, rapid oscillation of state-determining variables [30,31]. In FFI the appetite for sleep is preserved, but true sleep cannot be consumed, and intrusions of REM sleep, which seems to be indispensable for the organism survival, are responsible for the behavioural manifestations of vivid dreams and oneirism.

The third report concerns a Mulvihill-Smith syndrome. Since its recognition by Mulvihill and Smith [44], nine patients have been reported, but only in two cases sleep features have been studied [12, 63]. The sleep abnormalities shown by the two patients are similar to those reported in FFI, Morvan's chorea and delirium tremens. There is almost complete lack of sleep lasting for weeks or months, absence of sleep spindles and K-complexes, complete loss of SWS, absence of muscle atonia during REM sleep.

The same authors reporting sleep disturbances in Mulvihill-Smith syndrome have described the presence of hallucinations in their patients. We cannot exclude that psychotic symptoms may represent, in this context, the intrusion of REM sleep into wakefulness.

Thus, it seems to be plausible that also this rare syndrome might be consequent to a severe and progressive dysfunction of the thalamo-limbic system, although pathological verifications are not available yet.

## 4. Conclusion

Also in physiologic conditions, mentation seems to be proper not only of wakefulness, as obvious, and of REM sleep, as it is known since the end of the Fiftieths, but also in NREM sleep. Other circumstances of overlap between states of being are present in nature both as physiologic and pathologic manifestations. The state-boundary dyscontrol becomes evident in some peculiar syndromes such as narcolepsy, RBD, status dissociatus and the most disrupted condition of it, named *agrypnia excitata*, which characterises the behavioural signs and symptoms of FFI, Mulvihill-Smith syndrome, as described above, and of Morvan's fibrillary chorea and delirium tremens.

Nevertheless, it must be underlined that isolated and often bizarre sleep-related events may occur also in healthy people without need of extensive investigations. Attention must instead be paid for those cases that, because of violent behaviours acted-out, could have forensic implications as profusely debated in the literature [16,32,34,36,37,41].

## References

[1] J. Antrobus, REM and NREM sleep reports: comparison of word frequencies by cognitive classes, *Psychophysiology* **20** (1983), 562–568.

[2] O. Bartsch, K.D. Tympner, E. Schwinger and R.J. Gorlin, Mulvihill-Smith syndrome: case report and review, *Journal of Medical Genetics* **31** (1994), 707–711.

[3] C. Bassetti and M.S. Aldrich, Narcolepsy, *Neurologic Clinics* **14** (1996), 545–571.

[4] B.F. Boeve, M. Silber, J. Parisi et al., Synucleinopathy pathology and REM sleep behaviour disorder plus dementia or parkinsonism, *Neurology* **61** (2003), 40–45.

[5] B.F. Boeve, M.H. Silber, C.B. Saper et al., Pathophysiology of REM sleep behaviour disorder and relevance to neurodegenerative disease, *Brain* **130** (2007), 2770–2788.

[6] M. Casagrande, C. Violani, F. Lucidi, E. Buttinelli and M. Bertini, Variations in sleep mentation as a function of time of night, *The International Journal of Neuroscience* **85** (1996), 19–30.

[7] S.L. Delinger, R. Patarca and J.A. Hobson, Differential enhancement of rapid eye movement sleep signs in the cat: a comparison of microinjection of the cholinergic agonist carbachol and the beta-adrenergic antagonist propranolol on pontogeniculo-occipital wave clusters, *Brain Research* **473** (1988), 116–126.

[8] W.C. Dement and N. Kleitman, Cyclic variations in EEG during sleep and their relations to eye movements, body motility, and dreaming, *Electroencephalography and Clinical Neurophysiology* **9** (1957), 673–690.

[9] W.C. Dement, The biological role of REM sleep (circa 1968), in: *Sleep Physiology and Pathology*, A. Kales, ed., Philadelphia: Lippincott, 1969, pp. 245–265.

[10] I. Feinberg and J.D. March, Observations on delta homeostasis, the one-stimulus model of NREM-REM alternation and the neurobiologic implications of experimental dream studies, *Behavioural Brain Research* **67** (1999), 121–131.

[11] S.M. Feldman and H.J. Waller, Dissociation of electrocortical activation and behavioral arousal, *Nature* **196** (1962), 1329–1322.

[12] R. Ferri, B. Lanuzza, F.I.I. Cosentino et al., Agrypnia excitata in a patient with progeroid short stature and pigmented Nevi (Mulvihill-Smith syndrome), *Journal of Sleep Research* **14** (2005), 463–470.

[13] M.F. Folstein, S.E. Folstein and P.R. McHugh, "Mini Mental State": a practical method for grading the cognitive state of patients for clinician, *Journal of Psychiatric Research* **12** (1975), 189–198.

[14] D. Foulkes, Non rapid eye movement mentation, *Experimental Neurology* **4** (1967), 28–38.

[15] J.B.E. Gélineau, De la narcolepsye, *Gazette Des Hôpitaux* **53** (1880), 626–628.

[16] C. Guilleminault, A. Moscovitch and D. Leger, Forensic sleep medicine: nocturnal wandering and violence, *Sleep* **18** (1995), 740–748.

[17] I. Gritti, M. Mariotti and M. Mancia, GABAergic and cholinergic basal forebrain and preoptic-anterior hypothalamic projections to the mediodorsal nucleus of the thalamus in the cat, *Neuroscience* **85** (1998), 149–178.

[18] J.W. Hastings, B. Rusak and Z. Boulos, Circadian rhytms: the physiology of biological timing, in: *Neural and Integrative Animal Physiology, Comparative Animal Physiology*, (4th ed.), C.L. Prosser, ed., New York: Wiley-Liss, 1991, pp. 435–546.

[19] J. Hazra, Effect of hemicholinium-3 on slow wave and paradoxical sleep of cat, *European Journal of Pharmacology* **11** (1970), 395–397.

[20] K. Henley and A.R. Morrison, A re-evaluation of the effects of lesions of the pontine tegmentium and locus coeruleus on phenomena of paradoxical sleep in cat, *Acta Neurobiologie Experimentalis* **34** (1974), 215–232.

[21] J.A. Hobson and R.W. McCarley, The brain as a dream state generator: an activation-synthesis hypothesis of the dream process, *The American Journal of Psychiatry* **134** (1977), 1335–1348.

[22] J.A. Hobson and R. Stickgold, Sleep. Sleep the beloved teacher? *Current Biology* **5** (1995), 35–36.

[23] N. Hofle, T. Paus, D. Reutens et al., Regional cerebral blood flow changes as a function of delta and spindle activity during slow wave sleep in humans, *The Journal of Neuroscience: the Official Journal of the Society for Neuroscience* **17** (1997), 4800–4808.

[24] M. Jouvet and F. Delorme, Locus coeruleus et sommeil paradoxal, *Comptes Rendus des Séances de la Société de Biologie et de Ses Filiales* **159** (1965), 895–899.

[25] M.H. Kryger, T. Roth and W.C. Dement, *Principles and Practice of Sleep Medicine*, Philadelphia: Saunders (1989).

[26] G.C. Lairy, L. Goldsteinas and A. Guennoc, Disturbances of sleep in patients with confusional and demential syndromes, *Electroencephalography and Clinical Neurophysiology* **23** (1967), 286.

[27] J. Lu, D. Sherman, M. Devor and C. Saper, A putative flip-flop switch for control of REM sleep, *Nature* **441** (2006), 589–594.

[28] E. Lugaresi, R. Medori, A. Baruzzi et al., Fatal familial insomnia and dysautonomia with selective degeneration of thalamic nuclei, *The New England Journal of Medicine* **315** (1986), 997–1003.

[29] E. Lugaresi and F. Provini, Agrypnia Excitata: clinical features and pathophysiological implications, *Sleep Medicine Reviews* **5** (2001), 313–322.

[30] M.W. Mahowald and C.H. Schenck, Status dissociatus – a perspective on states of being, *Sleep* **14** (1991), 69–79.

[31] M.W. Mahowald and C.H. Schenck, Dissociated states of wakefulness and sleep, *Neurology* **42**(Suppl 6) (1992), 44–52.

[32] M.W. Mahowald and C.H. Schenck, Complex motor behavior arising during the sleep period: forensic science implications, *Sleep* **18** (1995), 724–727.

[33] M.W. Mahowald and C.H. Schenck, Evolving concepts of human state dissociation, *Archives Italiennes de Biologie* **139** (2001), 269–300.

[34] M.W. Mahowald and C.H. Schenck, REM sleep behaviour disorder, in: *Principles and Practice of Sleep Medicine*, M. Kryger, T. Roth and W. Dement, eds, Elsevier/Saunders, Philadelphia, 2005, pp. 724–741.

[35] M.W. Mahowald and C.H. Schenck, Insights from studying human sleep disorders, *Nature* **437** (2005), 1279–1285.

[36] M.W. Mahowald, S.R. Bundlie, T.D. Hurwitz and C.H. Schenck, Sleep violence-forensic sciences implications: polygraphic and video documentation, *Journal of Forensic Sciences* **35** (1990), 413–432.

[37] M.W. Mahowald, C.H. Schenck and M.A. Cramer Bornemann, Sleep-related violence, *Current Neurology and Neuroscience Reports* **5** (2005), 153–158.

[38] D.J. McGinty, Somnolence, recovery and hyposomnia following ventro-medial diencephalic lesions in the rat, *Electroencephalography and Clinical Neurophysiology* **26** (1969), 70–79.

[39] E. Mignot, Genetic and familial aspects of narcolepsy, *Neurology* **50** (1998), S16–S22.

[40] M.M. Mitler and W.C. Dement, Cataplectic-like behavior in cats after microinjection of carbachol in pontine reticular formation, *Brain Research* **69** (1974), 335–343.

[41] H. Moldofsky, R. Gilbert, F.A. Lue and A.W. MacLean, Sleep-related violence, *Sleep* **18** (1995), 731–739.

[42] P. Montagna, Fatal familial insomnia: a model disease in sleep physiopathology, *Sleep Medicine Reviews* **9** (2005), 339–353.

[43] P. Montagna and E. Lugaresi, Agrypnia Excitata: a generalized overactivity syndrome and a useful concept in the neurophysiopathology of sleep, *Clinical Neurophysiology: Official Journal of the International Federation of Clinical Neurophysiology* **113** (2002), 552–560.

[44] J.J. Mulvihill and D.W. Smith, Another disorder with prenatal shortness of stature and premature aging, *Birth Defects Original Article Series* **11** (1975), 368–371.

[45] W.J. Murray, A new method for measuring daytime sleepiness: the Epworth sleepiness scale, *Sleep* **14** (1991), 540–545.

[46] T.A. Nielsen, A review of mentation in REM and NREM sleep: "Covert" REM sleep as a possible reconciliation of two opposing models, *The Behavioral and Brain Sciences* **23** (2000), 851–866.

[47] S. Nightingale, J.C. Orgill, I.O. Ebrahim et al., The association between narcolepsy and REM behaviour disorder (RBD), *Sleep Medicine* **6** (2005), 253–258.

[48] S. Nishino, B. Ripley, S. Overem, G.J. Lammers and E. Mignot, Hypocretin (orexin) deficiency in human narcolepsy, *Lancet* **355** (2000), 39–40.

[49] T. Pivik and D. Foulkes, NREM mentation: relation to personality, orientation time, and time of night, *Journal of Consulting and Clinical Psychology* **32** (1968), 144–151.

[50] F. Provini, C. Lombardi and E. Lugaresi, Insomnia in neurological diseases, *Seminars in Neurology* **25** (2005), 81–89.

[51] A. Raggi, D. Perani, G. Giaccone et al., The behavioural features of fatal familial insomnia: a new Italian case with pathological verification, *Sleep Medicine* **10** (2009), 581–585.

[52] G.S. Richardson, M.A. Carskadon, W. Flagg, J. Van den Hoed, W.C. Dement and M.M. Mitler, Excessive daytime sleepiness in man: multiple sleep latency measurement in narcoleptic and control subjects, *Electroencephalography and Clinical Neurophysiology* **45** (1978), 621–627.

[53] V.S. Rotenberg, The estimation of sleep quality in different stages and cycles of sleep, *Journal of Sleep Research* **2** (1993), 17–20.

[54] C.H. Schenck, S.R. Bundlie, M.G. Ettinger and M.W. Mahowald, Chronich behavioural disorders of human REM sleep: a new category of parasomnia, *Sleep* **9** (1986), 293–308.

[55] M.E. Seligman and A. Yellen, What is a dream? *Behaviour Research and Therapy* **25** (1987), 1–24.

[56] E. Stotz-Potter and E. Benarroch, Removal of GABAergic inhibition in the mediodorsal nucleus of the rat thalamus leads to increase in heart rate and blood pressure, *Neuroscience Letters* **247** (1998), 127–130.

[57] S. Taheri, J.M. Zeitzer and E. Mignot, The role of hypocretins (orexins) in sleep regulation and narcolepsy, *Annual Review of Neuroscience* **25** (2002), 283–313.

[58] P. Tinuper, P. Montagna, R Medori et al., The thalamus participates in the regulation of the sleep-waking cycle: a clinico-pathological study in fatal familial thalamic degeneration, *Electroencephalography and Clinical Neurophysiology* **73** (1989), 117–123.

[59] J.L. Velayos, M. Oliva and F. Alfageme, Afferent projections to the mediodorsal and anterior thalamic nuclei in the cat. Anatomical-clinical correlations, *Brain Pathology* (*Zurich, Switzerland*) **8** (1998), 549–552.

[60] J. Villablanca and M.E. Salinas-Zeballos, Sleep-wakefulness, EEG and behavioral studies of chronic cats without the thalamus: the "athalamic" cat, *Archives Italiennes de Biologie* **110** (1972), 383–411.

[61] H.H. Webster and B.E. Jones, Neurotoxic lesions of the dorsolateral pontomesencephalic tegmentum-cholinergic cell area in the cat, II. Effects upon sleep-waking states, *Brain Research* **458** (1998), 285–302.

[62] D. Wechsler, *WAIS-R Manual*, the Psychological Corporation, San Antonio, TX, 1981.

[63] T. Yagihashi, M. Kato, K. Izumi et al., Case report: Adult phenotype of Mulvihill-Smith syndrome, *American Journal of Medical genetics. Part A* **149A** (2009), 496–500.
